# Six-month field efficacy and safety of the combined treatment of dogs with Frontline Tri-Act® and NexGard Spectra®

**DOI:** 10.1186/s13071-018-2957-7

**Published:** 2018-07-16

**Authors:** Jessica M. Abbate, Ettore Napoli, Francesca Arfuso, Gabriella Gaglio, Salvatore Giannetto, Lenaig Halos, Frederic Beugnet, Emanuele Brianti

**Affiliations:** 1 Dipartimento di Scienze Veterinarie, Università degli Studi di Messina, Polo Universitario Annunziata, 98168 Messina, Italy; 2 Boehringer-Ingelheim Animal Health, 29 Av Tony Garnier, Lyon, France

**Keywords:** Afoxolaner/milbemycin oxime, Canine vector-borne diseases, Ectoparasites, Efficacy, Fipronil/permethrin, Nematodes, Safety

## Abstract

**Background:**

Safety and efficacy of the combined monthly use of spot-on fipronil 6.76% w/v / permethrin 50.48% w/v (Frontline Tri-Act®) and chewable tablets of afoxolaner 1.9% w/w / milbemycin oxime 0.4% w/w (NexGard Spectra®) in dogs was evaluated in a field study over a period of 6 months.

**Methods:**

Forty-one healthy dogs living in highly endemic area for canine leishmaniosis and other canine vector borne diseases (VBD) were included in the study at the beginning of the *Leishmania* transmission season. Sixteen dogs were pet dogs living each in a single household; twenty-five dogs were hunting dogs living in three kennels. At inclusion, the dogs were ELISA (rapid test) negative for antibodies to *Anaplasma*, *Borrelia*, *Ehrlichia*, and for antigens of *Dirofilaria*. The dogs were also negative for blood microfilariae at the Knott’s test, and no clinical or haematological abnormalities were observed. Of the included dogs, six hunting, apparently healthy, dogs were ELISA (rapid test) positive to *Leishmania*, and some were naturally infected by gastrointestinal nematodes (58.5%) and/or infested by fleas (58.5%) and ticks (9.8%). All the included dogs were treated at Days 0, 28, 56, 84, 112 and 140, and followed-up for efficacy until the study end (Day 168).

**Results:**

No adverse events related to the two products, nor skin reactions, general signs, or changes in the haematological profile, were observed during the study. At Day 14, anthelminthic efficacy was 100% for *Toxocara canis*, *Toxascaris leonina* and *Capillaria aerophila*, while few hunting dogs were still shedding eggs of *Trichuris vulpis* (1/25 hunting dog) and Ancylostomatidae (9/25 hunting dogs). All pet dogs were nematode free at the end of the study. Hunting dogs were free of roundworms and whipworms. Twenty-four hours after the first treatment, 95.8% of the ectoparasite infested dogs were free from fleas and ticks. Ectoparasites were significantly controlled during the 6-month study period, with 100% efficacy on both fleas and ticks from Day 56 to Day 168. Blood and serum samples collected on Day 168 were tested for vector-borne pathogens using same methods of the inclusion and no new seroconversions or circulating blood microfilariae were observed.

**Conclusions:**

Concomitant use of Frontline Tri-Act® and NexGard Spectra® in dogs for six months was well tolerated. The combination was effective in controlling fleas, ticks, gastro-intestinal nematodes, and neither new seroconversion to the tested vector-borne pathogens nor blood microfilariae were detected in treated dogs at the end of the study.

## Background

Dogs are continuously exposed to parasitic infections and vector-borne diseases (VBD), with some of them being of zoonotic concern [1, 2]. The risk is higher in animals living in communities (e.g. kennels or shelters) and/or with access to the outdoors [3]. Despite better attention to canine health and the use of highly effective anti-parasitic products, recent studies conducted throughout Europe have shown that intestinal nematode infections remain a common occurrence in dogs [4–6]. A recent survey conducted on 1390 owned dogs reported that more than a third of them were shedding eggs/cysts of endoparasites, i.e. nematodes, cestodes and/or protozoans [7]. The ratio of endoparasitic infections is higher if kennel dogs are included in the epidemiological surveys [8].

Ectoparasite infections, mainly caused by fleas and ticks, constantly challenge dogs [9]. Their role as vectors of many pathogens highlights the need for an efficacious year-round strategy of control [10, 11]. Canine leishmaniosis (CanL), caused by *Leishmania infantum*, and canine dirofilariosis, due to *Dirofilaria immitis* or *Dirofilaria repens*, are among the most diffused and common VBD of dogs throughout Europe [12–15]. Several drivers are currently influencing the epidemiology of these diseases. Changes in vector presence, abundance and activity, as well as increased movements of animals from endemic areas into free territories and *vice versa*, seem to play key roles in this change in epidemiology [16]. As a result, the current distribution of CanL and canine heartworm disease (CHD) due to *D. immitis* is overlapping in several European countries, including Albania [17], Greece [18], France [2], Italy [3], Portugal [13, 14] and Spain [19]. This means that dogs living in these areas are at risk for both diseases during at least 4–6 months per year on average, which corresponds to the period of activity of the respective vectors (i.e. sand flies and mosquitoes).

Advances in research for the control of endo- and ectoparasites have resulted in the development of several highly effective veterinary products for the prevention of canine VBD. However, a single product that guarantees prevention against endoparasites, ectoparasites and canine VBD is not yet available. Therefore, the combined use of different drugs is likely to represent the only preventative solution to protect dogs under particular epidemiological settings [20].

Recently, two new antiparasitic products, namely Frontline Tri-Act® (Merial, now part of Boehringer Ingelheim), a topical solution combination of fipronil 6.76% w/v and permethrin 50.48% w/v, and NexGard Spectra® (Merial, now part of Boehringer Ingelheim), a chewable tablet containing afoxolaner 1.9% w/w and milbemycin oxime 0.4% w/w, have been launched onto the veterinary market. The former is a spot-on formulation, effective for treatment and prevention of flea infestations, including *Ctenocephalides felis* and *Ctenocephalides canis* [21, 22] and ticks, including *Dermacentor reticulatus*, *Rhipicephalus sanguineus* (*sensu lato*) and *Ixodes ricinus* [23, 24]*.* The product has shown a repellent and insecticidal activity against mosquitoes and stable flies [25, 26], and against phlebotomine sand flies, the vectors of CanL, under laboratory conditions [27]. Moreover, the efficacy in preventing CanL in a cohort of naturally exposed dogs has been recently demonstrated in a hyperendemic area of Greece [18]. NexGard Spectra® is a systemic insecticidal and acaricidal product in a chewable formulation licensed for treatment and prevention of ectoparasite infestations (fleas and ticks), and treatment of gastrointestinal nematodes [7]. This product, due to the presence of milbemycin oxime, is also effective for the prevention of CHD [28].

The concomitant use of these two products could represent a reliable solution for a comprehensive protection against both endo- and ectoparasites, including the prevention of CanL and CHD. Currently, no information on their concomitant administration is available in literature. Therefore, the aim of the present study was to assess the safety and the efficacy of the monthly concurrent treatments with Frontline Tri-Act® and NexGard Spectra® in dogs for a period of six months.

## Methods

### Study design

The study was a non-controlled, un-blinded, field trial conducted on privately owned dogs. Animals were included in the study after collection of the owner written informed consent. The study was conducted in Sicily and Calabria regions (southern Italy) from May 2016 to January 2017.

Seventy-four dogs with a minimum body weight of 2 kg and older than 8 weeks were screened at the pre-inclusion visit on study day -7 (Day -7). Each animal was physically examined, and individual faecal and blood samples were collected for analyses (see Sample analyses section). Only dogs with satisfactory general health condition, not infected by agents of chronic debilitating diseases or VBD (i.e. anaplasmosis, borreliosis, ehrlichiosis and leishmaniosis), and without circulating microfilariae, were enrolled.

On Day 0, 41 dogs were included in the study and sampled again for faeces, checked for the presence of ectoparasites, and simultaneously treated with the two testing products at therapeutic doses and in compliance with manufacturer’s instructions. Frontline Tri-Act® pipette was applied directly on animal’s skin and divided in two approximately equal spots (i.e. at the base of the skull and between the blades of shoulders). NexGard Spectra® was administered to dogs initially by offering the chew for spontaneous ingestion or by putting it directly into the mouth, if the dog had not eaten the drug spontaneously. After treatment, each dog was observed for 2 h in order to detect any side effects (e.g. ptyalism, vomiting or skin reactions). All enrolled animals remained with their owners and kept as per normal routine for the entire study. The owners were instructed to assess the animal’s health status daily and to notify any adverse event or behaviour abnormalities immediately.

After initial Day 0 treatment, the dogs were followed-up on Days 1, 14, 28, 56, 84, 112, 140 and 168, and were retreated with the two testing products every 28 days (± 2) after clinical check and sampling (Table 1). At each visit the dogs were clinically examined by evaluation of body temperature, body weight, nutritional status, respiratory, cardiovascular and gastrointestinal systems, and checked for tick and flea presence by thumbing and combing the fur for ~3 min. Individual faecal samples were collected and screened for gastrointestinal parasites using copromicroscopical analyses at each visit. Blood samples were collected from each dog on Days -7, 28, 84 and 168.

**Table 1:** Dates and scheduled operations

Activity	Pre-inclusion (Day -7)	Inclusion (Day 0)	Day 1 visit	Day 14 visit	Day 28 visit	Day 56 visit	Day 84 visit	Day 112 visit	Day 140 visit	Day 168 visit
Clinical examination	**+**	**+**	**+**	**+**	**+**	**+**	**+**	**+**	**+**	**+**
CBC	**+**				**+**		**+**			**+**
Copromicroscopy (McMaster + Baermann)	**+**	**+**		**+**	**+**	**+**	**+**	**+**	**+**	**+**
Blood microfilariae	**+**									**+**
Ectoparasites	**+**	**+**	**+**	**+**	**+**	**+**	**+**	**+**	**+**	**+**
*Anaplasma* spp. serology	**+**									**+**
*Ehrlichia canis* serology	**+**									**+**
*Leishmania infantum* serology	**+**									**+**
Treatment (Frontline Tri-Act+NexGard Spectra)		**+**			**+**	**+**	**+**	**+**	**+**	

### Sample analyses

Faecal samples were processed with Baermann-Wetzel technique [29] for the detection of broncho-pulmonary larvae and by a modified McMaster technique with a cut-off of 15 eggs per gram (epg) [30–33].

Blood was collected from a peripheral vein (jugular or cephalic) using a standard technique and stored in 2.5 ml anticoagulant (K_3_ EDTA) tube and in 2.5 ml cloth activator tube. A complete cells blood count (CBC), including red blood cells (RBC), haemoglobin (HGB), haematocrit (HCT), white blood cells (WBC) and platelets (PLT), was performed on all blood samples using an automated haematology analyser (Benesphera **H32 VET, Avantor Performance Materials Inc., Center Valley, PA, USA).

The presence of circulating microfilariae was assessed using a modified Knott’s test [34] on Days -7 and 168. Observed microfilariae were identified to species level using morphometric criteria [34].

Sera samples collected on Days -7 and 168 were tested by rapid ELISA assays for *L. infantum* using the Canine *Leishmania* Antibody Test Kit produced by IDEXX® (IDEXX laboratories, Westbrook, ME, USA), and for *Anaplasma* spp., *Borrelia* spp., *Ehrlichia* spp., and *D. immitis* using SNAP® 4Dx Plus (IDEXX laboratories, Westbrook, ME, USA) following the producer's recommendations.

### Safety assessments

Data on clinical parameters (body temperature, body weight, nutritional status, respiratory, cardiovascular and gastrointestinal systems) and CBC collected during clinical examinations and from analyses were compared to the data collected at the pre-inclusion visit, which were considered as baseline values. Pre-treatment haematological blood parameters (Day -7) were compared with values obtained at Days 28, 84 and 168 by means of one-way analysis of variance (ANOVA) for repeated measures.

The same statistical analysis was performed in order to evaluate the effect of time on dog’s body weight values. When significant differences were found, Bonferroni’s post hoc comparison was applied. A *P*-value < 0.05 was considered statistically significant. The statistical analysis was performed using the software GraphPad Prism version 5.1 (GraphPad Software, San Diego, California, USA).

Data on local tolerance of the spot-on treatment were also collected and the application sites were particularly examined for changes of the hair coat and skin.

### Efficacy assessments

Efficacy against endo- and ectoparasites was evaluated in the animals that scored positive at the inclusion. The effect (curative or preventive) against ectoparasites was assessed 24 h after the initial treatment, at Day 14, and then monthly until Day 168. The efficacy is expressed as a percentage of flea or tick free dogs as no ectoparasite counts were performed.

Due to the poor correlation between the egg numbers and parasite infestation for nematodes in dogs, the number of infected dogs at each sampling time-point assessed the efficacy against gastrointestinal and respiratory nematodes. The percentage of nematode free dogs was calculated for each parasite species.

## Results

### Enrolment

Among the 74 dogs screened, 41 dogs, with a mean age of 3.6 years and a weight ranging from 3.4 to 32 kg, fulfilled the inclusion criteria and were included in the study at Day 0 (Table 2). The 33 excluded dogs had abnormal haematological parameters or tested positive to CanL or other vector borne diseases (Table 3). Six of the included hunting dogs were, however, seropositive to *Leishmania* but did not show clinical signs or pathological alterations and, therefore, were kept included because co-housed in the same pen with other enrolled dogs.

**Table 2:** Characteristics of the 41 dogs included in the study

Variable	No. of dogs
Age	
≤12 months	14
12–60 months	15
> 60 months	12
Size	
Small (≤ 7 kg)	4
Medium (7-18 kg)	24
Large (> 18 kg)	13
Hair coat	
Short	28
Medium	7
Long	6
Life style	
Pet	16
Hunting	25
Sex	
Female	19
Male	22
Breed	
Cross-breed	21
Pure-breed	20

**Table 3:** Clinical assessment and evaluation of the 74 dogs screened at the pre-inclusion visit

*Leishmania infantum* seropositive dogs	25/74 (33.8%)
*Ehrlichia canis* seropositive dogs	2/74 (2.7%)
*Anaplasma* spp. seropositive dogs	1/74 (1.4%)
Gastrointestinal parasite positive dogs	29/74 (39.2%)
Fleas positive dogs	26/74 (35.1%)
Ticks positive dogs	4/74 (5.4%)
Alterations of the haematological parameters	19/74 (25.7%)
Owners not complying with the study protocol	2/74 (2.7%)

Of the 41 enrolled dogs, 16 were pet dogs living each in a single household, and 25 were hunting dogs living in three kennels (6 in kennel 1, 7 in kennel 2, and 12 in kennel 3). One dog was lost by the owner after Day 84, one hunting dog deceased as outcome of hierarchy aggression by another dog (after Day 112), two other dogs did not come for veterinary visit and sampling on Days 84, 112, and 140 because of owner’s unavailability, but came back on Day 168. For statistical analysis, only complete dog cases at Day 168 (*n* = 37) were used.

### Safety

Neither adverse events related to the two products, nor skin reactions or general signs were observed during the study. No change of clinical examination parameters (i.e. body temperature, nutritional status, heart rate and respiratory rate) was recorded. The mean body weight showed a non-significant slight increase from the baseline, i.e. from 16.1 to 17.3 kg (∆ = 1.22, *P* = 0.949).

Mean values of haematological parameters were within physiological limits of the species throughout the study period. However, a statistical significant increase of HCT (*F*_(3, 160)_ *=* 36.21, *P* < 0.0001), HGB (*F*_(3, 160)_ *=* 44.35, *P* < 0.0001) and PLT (*F*_(3, 160)_ *=* 3.08, *P* = 0.029) compared to the baseline values was observed on Days 84 and 168 (Table 4).

**Table 4:** Mean values ± standard deviation (M ± SD) of haematological parameters recorded in dogs during the study. Dogs were treated with Frontline Tri-Act and Nexgard Spectra on study Day 0 and thereafter every 28 (± 2 days) up to Day 140

Parameters	RR^a^	Study days				*P-*value
		Day -7	Day 28	Day 84	Day 168	
		M ± SD	M ± SD	M ± SD	M ± SD	
RBC	5.5–8.5 × 10^6^/μl	6.8 ± 0.9	7.0 ± 0.8	7.2 ± 0.7	7.1 ± 0.4	0.12
WBC	6–17 × 10^3^/μl	12.9 ± 2.9	12.0 ± 2.5	12.1 ± 2.3	11.9 ± 12.6	0.07
HCT	39–56%	42.2 ± 6.2	43.3 ± 4.8	46.2 ± 4.4*	52.4 ± 4.2*	< 0.001
HGB	11–19 g/dl	14.3 ± 2.2	14.42 ± 1.5	14.9 ± 1.4	18.3 ± 2.2*	< 0.001
PLT	117–460 × 10^3^/μl	231.6 ± 84.1	233.6 ± 75.3	244.1 ± 96.7	284.8 ± 108.2*	< 0.05

*Abbreviations*: *RBC* Red blood cells, *WBC* white blood cells, *HCT* haematocrit, *HGB* haemoglobin, *PLT* platelets count

*Statistically significant different *vs* Day -7

^a^Reference range [36]

### Efficacy assessments

At Day 0, 24 dogs (58.5%) were found infected with gastrointestinal nematodes, i.e. 18.8% (3/16) pet dogs and 84% (21/25) hunting dogs (*χ*^2^ = 17.1137, *df* = 1, *P* < 0.0001). At inclusion, six parasite species/taxa were identified, with *Trichuris vulpis* (20/41, 48.8%) and Ancylostomatidae (15/41, 36.6%) as the most frequent species, followed by *Capillaria aerophila* (8/41, 19.5 %), *Toxascaris leonina* (7/41, 17.1%), *Toxocara canis* (6/41, 14.6%) and *Dipylidium caninum* (3/41, 7.3%).

At Day 14 visit, no eggs of *C. aerophila*, *T. canis* and *T. leonina* were found at coproscopy, while some hunting dogs were still shedding eggs of *T. vulpis* (1/25) and Ancylostomatidae (9/25). All pet dogs were found nematode free from Day 14 to Day 168. Except for the Ancylostomatidae, all the hunting dogs were nematode free from Day 112 assessment up to the end of the study (Table 5). None of the enrolled dogs tested positive at the Baermann-Wetzel technique neither at the pre-inclusion nor throughout the study period.

**Table 5:** Number and percentage of infested dogs examined on pre- and post-treatment days

Study days	Number of positive dogs per endoparasite species (%)					
	*Trichuris vulpis*	Ancylostomatidae	*Capillaria aerophila*	*Toxocara canis*	*Toxascaris leonina*	*Dipylidium caninum* ^a^
Day 0	20/41 (48.8)	15/41 (36.6)	8/41 (19.5)	6/41 (14.6)	7/41 (17.1)	3/41 (7.3)
Day 14	1/41 (2.4)	9/41 (22.0)	0/41 (0)	0/41 (0)	0/41 (0)	0/41 (0)
Day 28	2/41 (4.9)	15/41 (36.6)	4/41 (9.8)	0/41 (0)	0/41 (0)	1/41 (2.4)
Day 56	1/41 (2.4)	15/41 (36.6)	0/41 (0)	0/41 (0)	0/41 (0)	0/41 (0)
Day 84	1/39 (2.6)	10/39 (25.6)	0/39 (0)	1/39 (2.6)	0/39 (0)	0/39 (0)
Day 112	0/37 (0)	12/37 (32.4)	0/37 (0)	1/37 (2.7)	0/37 (0)	0/37 (0)
Day 140	0/37 (0)	13/37 (35.1)	0/37 (0)	0/37 (0)	0/37 (0)	0/37 (0)
Day 168	0/37 (0)	10/37 (27)	0/37 (0)	0/37 (0)	0/37 (0)	0/37 (0)

^a^Dogs found infected with *Dipylidium caninum* were treated with praziquantel, since this tapeworm species is not targeted by the two products tested in the study

On Day 0 visits the percentage of dogs infested by fleas was 58.5% (3/16 pet dogs and 21/25 hunting dogs; *χ*^2^ = 17.1137, *df* = 1, *P* < 0.0001). On Day 1, 40/41 (97.6%) of the dogs were flea-free and none of them was found infested by fleas on the following scheduled controls, except on Day 28 when seven hunting dogs (28%) scored flea positive (Table 6).

**Table 6:** Number of infested dogs and percentages of ectoparasite free dogs in the scheduled follow-ups. Dogs were treated with Frontline Tri-Act and Nexgard Spectra on study Day 0 and thereafter every 28 (± 2 days) up to Day 140

	Study days									
	Day -7	Day 0	Day 1	Day 14	Day 28	Day 56	Day 84	Day 112	Day 140	Day 168
Fleas *n/*total (% free dogs)	20/41 (51.2)	24/41 (41.5)	1/41 (97.6)	0/41 (100)	7/41 (83.0)	0/41 (100)	0/39 (100)	0/37 (100)	0/37 (100)	0/37 (100)
Ticks *n*/total (% free dogs)	1/41 (97.6)	4/41 (90.2)	1/41 (97.6)	0/41 (100)	0/41 (100)	0/41 (100)	0/39 (100)	0/37 (100)	0/37 (100)	0/37 (100)

On Day 0, tick infestation was observed in 1/16 (6.2%) pet dogs and 3/25 (12%) of the hunting kennel dogs; 24 h post-treatment the pet dog was still infested by ticks, while none of the animals scored positive for ticks in the other follow-ups (Table 6).

At inclusion and at Day 168, none of the enrolled dogs scored positive for *Anaplasma* spp., *Borrelia* spp., *Ehrlichia* spp. and *D. immitis* using the SNAP® 4Dx Plus test. No microfilariae were detected in blood samples, and all dogs scored ELISA (rapid test) negative to *L. infantum* antibodies with the exception of the six that were already positive at the inclusion.

## Discussion

This study demonstrates how the concurrent use of Frontline Tri-Act® and Nexgard Spectra® in dogs for six months is effective and well tolerated. These findings are largely in line with what has been previously observed with the single use of both products under laboratory and field conditions [7, 18, 22, 24, 35].

Haematological parameters remained within the reference ranges [36], with a significant increase of HCT, HGB and PLT observed on Days 84 and 168. This could be attributable to a general improvement of health conditions in treated dogs as they were dewormed and freed from ectoparasites. Also, it could be the result of increased physical activities [37], as the majority of these animals were hunting dogs and the last part of the study overlapped with the hunting season.

The species of gastrointestinal nematodes identified in the present field trial match with those reported in other surveys conducted throughout Europe [7]. The overall frequency of intestinal parasite infections reported here (from 14.6% to 48.8% according to species/taxa) was considerably high, especially in hunting dogs living in kennels with 84% of mixed infestation by endo- and ectoparasites. This underlines, once again how owned dogs may be significantly infected by intestinal parasites even if adult and apparently healthy pets [38]. All dogs included, whipworms and hookworms were the species with the highest frequencies (48.8 and 36.6%, respectively). The presence of *C. aerophila* in 8/41 (19.5%) of the dogs, seven hunting dogs and one pet dog, is of particular interest. This trichuroid nematode infects the lungs of domestic (i.e. dogs and cats) and wild carnivores (e.g. foxes) and may represent a zoonotic hazard [39, 40]. Capillariosis in dogs and cats is increasingly diagnosed throughout Europe and the frequency of infection reported here is the highest ever reported in dogs so far [5, 41, 42], confirming how certain animal categories (e.g. hunting dogs) may be at great risk of infection.

The efficacy against intestinal parasites reported in the present study largely matches that observed previously in an extensive European multicentre field study [7]. At Day 112 and after, all dogs were free of whipworms, roundworms, and *Capillaria*. Conversely, the efficacy against hookworms was lower to what has previously been observed under both natural and experimental conditions [7, 43]. It is important, however, to point out that all the dogs, which were continuously shedding Ancylostomatidae eggs, were hunting kennel dogs living with other untreated dogs in highly contaminated environment. Egg shedding would be related to re-infections, but also to regular coprophagy and passive migration of eggs from infected faeces. It is likely that re-infections could not be prevented due to the epidemiological situation in the three kennels. In kennel 1, the only hookworm infected dog became negative, in kennel 2, 4/7 infected dogs remained infected, and in kennel 3, from 8/12 at the beginning, 6/10 dogs were infected at the end of the study. Indeed, the pre-patent period of *Ancylostoma* can be as short as 14 days, and their control in kennels may require regular anthelmintic treatments administered every 2 weeks until control is obtained, and then monthly administrations [38, 44].

In the present trial fleas and ticks were efficiently controlled in treated animals starting from 24 hours post-treatment when 97.6% of flea and tick pre-infested animals were cured. Tick infestations were totally prevented as no ticks were found on dogs treated from Day 14 up to Day 168. On Day 28, a few hunting dogs (7/25) were found infested by fleas, but, interestingly, the majority of fleas collected during this visit were dead or moribund (data not shown). Thereafter, all dogs were flea-free. As already mentioned, the majority of dogs included in this trial were hunting dogs housed in kennels where other not included infested dogs were present, thus indicating a high regular exposure. Although acting with a different mode of action, both products here tested claim curative and preventative efficacy against ectoparasites of dogs; therefore, a synergistic action between them could not be ruled out and it may provide clues for the high efficacy profile here observed. However, it is important to note that the combined use of these products was primarily intended to provide a simultaneous protection against some VBD, especially CanL and CHD [7, 10, 24].

Despite this study was primarily designed to assess the safety and efficacy of the combined used of the two tested products, it also allowed the collection of supplementary data on the incidence of some vector-borne pathogens (VBP) in treated dogs. The study was indeed conducted in a hyper-endemic area for CanL, where a 54.6% incidence rate of infection has been reported in unprotected shelter dogs [45]. Most of the dogs screened at Day -7 were not enrolled in the study because they were already infected or diseased by *L. infantum* (see Table 3). The selected study area is also endemic for VBP transmitted by ticks, i.e. *Anaplasma phagocytophilum*, *Babesia canis*, *Ehrlichia canis* and *Rickettsia conorii* [46] and canine dirofilarioses, which have been reported in the same animal population [47, 48]. In the present trial dogs were enrolled in May and completed the study in December after they had been exposed to one entire CanL transmission season. Despite the high risk of transmission, no negative treated dogs scored seropositive to *L. infantum* at study end; analogously, no seroconversion against other VBD causing pathogens (i.e. *Anaplasma* spp., *Borrelia* spp., *Ehrlichia* spp. and *D. immitis*) or blood microfilariae were detected in dogs at the end of the study. Tough the limitations in terms of sensibility of the sole use of serological tests for the diagnosis of some VBP such as *L. infantum* (e.g. exposed/infected dogs that may have not seroconvert yet), results of the current trial further corroborate what has been observed in a previous study specifically designed to assess the efficacy of the association permethrin/fipronil in reducing the risk of transmission in naturally exposed dogs living in a CanL hyper-endemic area [18].

## Conclusions

The present study showed that the simultaneously administration of Frontline Tri-Act® and Nexgard Spectra® in dogs during a 6-month period is safe and provides a high efficacy in controlling ecto- and endoparasite infestations. In addition, neither new seroconversion to *Anaplasma* spp., *Borrelia* spp., *Ehrlichia* spp., *L. infantum* and *D. immitis* nor blood microfilariae were detected in treated dogs at the end of the study. By virtue of the repellent effect of the association fipronil/permethrin (Frontline Tri-Act®) against sand flies, and thanks to the efficacy of afoxolaner/milbemycin oxime (Nexgard Spectra®) in preventing canine heartworm disease, the concomitant use of these products offers a broad spectrum of protection which may be regarded as a safe and effective prevention strategy for dogs, particularly those living in or travelling to geographical areas where the risk of CanL and CHD is overlapping.

## Abbreviations

CanL: Canine leishmaniosis
CBC: Cells blood count
CHD: Canine heartworm disease
ELISA: Enzyme-linked immunosorbent assay
HCT: Haematocrit
HGB: Haemoglobin
PLT: Platelets
RBC: Red blood cells
VBD: Vector-borne diseases
VBP: Vector-borne pathogens
WBC: White blood cells
